# Sociodemographic influences on Student Mental Health and their association with activation-regulating functional impairments

**DOI:** 10.1371/journal.pone.0342731

**Published:** 2026-03-18

**Authors:** Mohammad Fraiwan, Fidaa Almomani, Hanan Hammouri

**Affiliations:** 1 Faculty of Computer and Information Technology, Jordan University of Science and Technology, Irbid, Jordan; 2 Department of Rehabilitation Sciences, Jordan University of Science and Technology, Irbid, Jordan; 3 Department of Mathematics and Statistics, Jordan University of Science and Technology, Irbid, Jordan; Ladoke Akintola University of Technology Teaching Hospital: LAUTECH Teaching Hospital, NIGERIA

## Abstract

Activation-regulating functions, grounded in Executive Function Theory, Self-Regulation Theory, and the Dysexecutive (DEX) Framework, refer to higher-order cognitive processes such as task initiation, sustained attention, planning, and prioritization that enable individuals to translate intentions into goal-directed action. In the university context, these functions are central to managing complex academic demands, organizing study activities, meeting deadlines, and maintaining engagement. Impairments in activation-regulating functions may increase stress, undermine motivation, and negatively affect mental health and overall well-being, particularly in the presence of persistent digital distractions such as smartphone use. This self-reported cross-sectional study involved 1204 participants and utilized the validated Arabic version of the activation dysexecutive questionnaire. Alongside this, demographic information and data on potential influencing factors were also collected. Significant differences were found, with various factors positively correlated with higher scores, indicating worse symptoms of activation dysfunction. Several of these factors are related to common Generation Z habits, such as the number of hours spent using smartphones or electronic devices (p<0.0001), number of weekly fast food consumption times (p<0.0001), number of social media platforms used (p=0.0027) and daily hours spent on social media (p<0.0001). Whereas, increasing number of weekly hours exercising/playing sports was correlated with lower dysfunction scores (p<0.0001). Other contributing factors include weak relationships with extended family (p=0.0008), specific family income groups (p=0.0099), specific GPA groups (p=0.0375), area of living (p=0.0023), previous consultations with a psychologist/psychiatrist (p<0.0001), and parental divorce (p<0.0406). On the other hand, participating in sports and exercising had a good impact on the activation subscale score (p<0.0001). So did having strong relationships with extended family (p=0.0008) and living with parents (p=0.0453). The study reveals significant factors affecting activation functions among university students, particularly Generation Z, with increased smartphone and social media use, frequent fast food consumption, weak familial relationships, and past mental health consultations linked to worse activation dysfunction. Conversely, participation in sports, strong family ties, and living with parents positively influence activation levels. To address these challenges, universities should promote healthier lifestyles through workshops on digital well-being and nutrition, encourage physical activity through organized sports, and provide resources for family counseling. Additionally, ensuring accessible mental health services can support students in navigating activation-related issues, fostering an environment conducive to both academic success and personal growth.

## Introduction

Activation-regulating functions, a subset of executive functions, are fundamental to young adults and university students as they shape their academic and personal development during this transitional stage of life. Defined as the cognitive processes that support task initiation, sustained attention, and goal-oriented behavior, activation-regulating functions are crucial for managing the complex demands of university life, including academic workloads, social interactions, and self-care routines [1,2]. For many young adults, ages 18–25, university is often the first environment where they are responsible for balancing numerous, often conflicting, priorities without the oversight they may have experienced at home or in structured secondary school settings [3].

In academic contexts, activation-regulating functions help students organize tasks, focus attention, and persist despite challenges, all of which are critical for academic performance [4]. When these functions are impaired, students may struggle with procrastination, poor time management, and difficulty adhering to study schedules, which can lead to increased stress and decreased academic success. Procrastination, for instance, is a well-documented issue linked to deficient activation-regulation, where students may delay starting tasks due to difficulty initiating action or sustaining focus [5].

Socially, young adults who can effectively regulate activation are better equipped to engage in meaningful interactions and manage relationships, which is significant given that university students often experience increased social autonomy. The ability to prioritize social interactions alongside academic responsibilities fosters a balanced lifestyle that supports mental well-being, an essential aspect given the high levels of stress reported among university students [6,7]. Furthermore, activation-regulating functions are associated with long-term goal setting and decision-making, skills that aid students in making choices about their future careers and personal aspirations [8]. Students who struggle with these functions may find it challenging to set and pursue long-term goals, potentially affecting career planning and academic pathways. Thus, supporting the development of these functions through targeted interventions, such as time management training and mindfulness practices, can significantly benefit young adults’ overall adaptation to university life and future career readiness [9].

Activation dysfunction can be measured using several standardized tools, each offering a unique perspective on cognitive, behavioral, and emotional aspects of activation regulation. The dysexecutive questionnaire (DEX), part of the behavioral assessment of the dysexecutive syndrome (BADS), is widely used to assess activation-related issues such as difficulties in initiating actions and planning; it incorporates self-reported and observer-rated evaluations, providing a comprehensive view of how activation issues manifest in everyday settings. The DEX has been shown to reliably assess activation dysfunction in clinical and research settings, highlighting its relevance for understanding executive function challenges among university students and young adults [10,11]. The behavior rating inventory of executive function-adult version (BRIEF-A) is another valuable tool that evaluates executive function domains like task initiation, capturing real-world activation difficulties such as procrastination and challenges in starting tasks; it has proven useful for both clinical and research purposes, including applications with university students [12,13]. For a performance-based approach, Conners’ continuous performance test (CPT) assesses sustained attention and impulse control, indirectly reflecting on activation dysfunction by identifying patterns in task initiation and sustained effort, which are important for maintaining focus and starting tasks [14].

## Materials and methods

### Ethical approval declarations

Approval for this study was obtained from the Institutional Review Board (IRB) at King Abdullah University Hospital and the Deanship of Scientific Research at Jordan University of Science and Technology in Jordan (Ref. 99/118/2023–1). All study procedures were conducted in accordance with the principles of the Declaration of Helsinki, and informed consent was collected from all participants before they joined the study.

### Design

This study employed a cross-sectional design with a descriptive-correlational approach to investigate factors related to symptoms of activation dysfunction in university students.

### Participants

The study’s participants were recruited from three public universities–Yarmouk University, Jordan University of Science and Technology (JUST), and Tafila Technical University–and two private institutions, Irbid University and Jadara University. While Yarmouk University, JUST, Irbid University, and Jadara University are located in northern Jordan, Tafila Technical University is situated in the south. Combined, these five universities have an estimated total student population of around 80,000.

### Sample

The study included a representative sample of students from all academic levels–freshmen, juniors, seniors, and final-year students–across the five universities. To determine the necessary sample size, a power analysis was conducted, estimating that 383 participants were required. This calculation ensured a 95% confidence level with a 5% margin of error.

### Procedure

After obtaining approval from the IRB and the Deanship of Research at JUST, the researchers selected large-sized classes (with over 80 students per section) for efficient data collection and improved response rates. With instructors’ consent, the researchers introduced the study to students, explaining its purpose and survey content. Surveys were distributed electronically via a QR code that directed students to a Google Forms survey. Students provided written consent by signing an informed consent form before submitting their responses. Approximately 5,000 students across the five universities were invited to participate in the electronic survey. The data was collected between April 1st, 2024 to December 30th, 2024.

The survey collected socio-demographic information such as age, gender, nationality, and residence type (rural or urban), along with family and social support details, including number of siblings and monthly family income. It also explored potential risk factors associated with dysexecutive functioning in relation to activation-regulating functions. These factors included body mass index (BMI), university type (private or public), academic level and major, GPA, frequency of part-time work, weekly exercise hours, daily smartphone use, weekly time spent on electronic games, number of social media accounts, daily social media use, weekly fast-food intake, and any previous consultations with psychiatrists or psychologists.

### Instrument

In this study, the validated Arabic version of the DEX revised (DEX-R) activation subscale was employed. The short Arabic version of the DEX-R demonstrated a Cronbach’s alpha of 0.94, indicating excellent internal consistency, along with strong test-retest reliability (ICC = 0.97). The activation subscale consists of a checklist of symptoms rated on a 5-point Likert scale, ranging from (1) “never” to (5)“very often.” This subscale possesses robust psychometric properties and is available in two formats: a self-report version, which was used in this research, and an independent-rating version. Initially, the dysexecutive questionnaire was developed as a supplementary tool for the behavioral assessment of dysexecutive syndrome (BADS) [16], aiming to identify observable changes in executive dysfunction following acquired brain injuries. It encompasses items that evaluate various facets of executive functioning, such as abstraction, impulsivity, confabulation, planning skills, mood regulation, and decision-making. The questionnaire addresses a wide array of specific concerns, including difficulties with attention, memory issues, information processing, behavioral control, emotion regulation, and self-awareness [17,18].

Originally designed as a qualitative assessment tool for customizing rehabilitation to individual challenges, the DEX has evolved into a quantitative tool for diagnostic purposes [19,20]. According to Simblett and Bateman [21], the DEX captures multiple latent variables that represent different dimensions of executive dysfunction. The focus of this study is on applying the activation subscale of the DEX-R within the Jordanian university population. A summary of the elements comprising this activation subscale from the DEX-R is presented in Table 1. The activation subscale was translated into Arabic, incorporating cultural adaptations to align with the nuances of the Arabic language. This translation process involved independent translation and back-translation by specialists who are proficient in English.

**Table 1:** Summary of the activation subscale elements from the DEX-R based on Stuss [15].

Item No. (DEX-R)	Item Name in (DEX-R)
3	Apathy problems
4	Initiation problems
7	Goal-neglect
11	Perseveration
12	Poor performance monitoring
23	Variable motivation
30	Restlessness, hyperkinesis
37	Poor decision-making ability

### Translation and reliability procedure

The activation subscale was translated into Arabic following established guidelines [17–19,22]. Three bilingual occupational therapists independently translated the DEX-R items into Arabic, resolving any discrepancies through discussion to reach a consensus on the final version. This version was then refined based on feedback from a pilot test with 65 participants. A back-translation was performed by a bilingual native English speaker from the United States, fluent in Arabic and residing in Jordan. A team of 10 researchers compared the original items with the back-translated items to assess similarity, using a cut-off score of 0.8 or higher (where 0 = “not similar” and 1 = “similar”) to evaluate translation adequacy. This threshold meant that at least 80% of evaluators agreed that the back-translated item closely matched the original, with scores below 0.8 indicating potential interpretation issues. In total, 97 participants aged 18–25 (mean age = 21.7 ± 2.9) took part in this cross-sectional study to assess subscale reliability. The sample, randomly selected over six months, included a variety of socioeconomic backgrounds to reflect the broader population distribution.

### Data processing and statistical analyses

**Fig 1:**
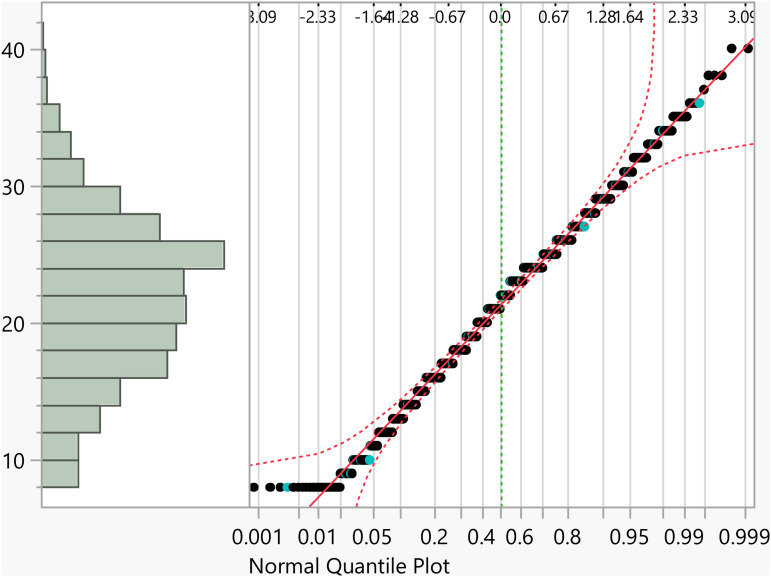
The Q-Q plot confirming the normal distribution of the activation subscale score.

Data analysis was conducted using JMP statistical software [23]. Initially, statistical summaries were generated for the dependent variable and key factors. For independent categorical variables, a t-test was used to compare means between two groups, and a one-way analysis of variance (ANOVA) was applied for variables with more than two groups. Tukey’s Honestly Significant Difference (HSD) test was then used to interpret significant differences (i.e., p<0.05). For continuous independent variables, linear regression analysis was conducted. These statistical methods required that the activation score (the outcome variable) follow a normal distribution, which was verified through quantile-quantile (Q-Q) plot, as shown in Fig 1.

## Results

Counts and percentages were calculated for the categorical variables, as detailed in Table 2 [24]. The demographic analysis shows that the majority of respondents are Jordanian (92%), with 61% living in urban areas. The gender distribution is nearly balanced, with 61% female and 39% male participants. Most respondents come from intact families (97%) and report strong connections with extended family (67%). The sample primarily includes students from public universities (92%), and a significant proportion (61%) belong to families earning less than 1,000 JOD monthly. Nearly all participants are undergraduates (99%), with the largest group being second-year students (51%). Academic performance varies, with 41% having a GPA in the B/B- range. Social media use is nearly universal at 99%, and a large majority (86%) live with their parents. A small fraction (7%) have consulted a psychologist or psychiatrist, and 64% do not engage in work while studying.

**Table 2:** Counts and percentages of categorical socio-demographic and possible risk factors. 1 JOD = 1.41 USD (fixed rate).

Variable	Value	Count	Percentage
Nationality	Other	93	8%
	Jordan	1111	92%
Area of living	Rural	467	39%
	City	737	61%
Sex	Male	466	39%
	Female	738	61%
Parents Divorced?	No	1168	97%
	Yes	36	3%
Strong relationship with extended family?	No	398	33%
	Yes	806	67%
University	Private	95	8%
	Public	1109	92%
Family monthly income (JOD [1])	<1000	731	61%
	1000-2000	333	28%
	2001-3000	70	6%
	>3000	70	6%
Undergraduate	Yes	1186	99%
	No	18	1%
Academic level (year)	1	97	8%
	2	612	51%
	3	294	24%
	4	201	17%
GPA	<C-	78	6%
	C,C-	270	22%
	B,B-	499	41%
	A,A-	357	30%
Work and Study?	Never	776	64%
	Sometimes	224	19%
	Most of the times	95	8%
	Always	109	9%
Do you have a social media account?	No	14	1%
	Yes	1190	99%
Do you live with parent(s)?	No	167	14%
	Yes	1037	86%
Previous visits to a psychologist/psychiatrist?	No	1116	93%
	Yes	88	7%

The mean and standard deviation (SD) for the continuous variables are presented in Table 3. These variables provide insights into lifestyle, physical health, and cognitive aspects. On average, participants have approximately 4.5 siblings (SD = 1.924), suggesting relatively large family sizes. Their average weight is 65.97 kg (SD = 15.03) and height is 167.48 cm (SD = 9.81), resulting in an average body mass index (BMI) of 23.39 (SD = 4.22), which is considered healthy. Participants report spending a considerable amount of time on social media daily, averaging 5.12 hours (SD = 4.11), and using smartphones or electronic devices for about 7.08 hours per day (SD = 4.30). They engage in physical exercise for approximately 3.8 hours weekly (SD = 5.51) but consume fast food around 2.1 times per week (SD = 1.44). Notably, their time spent on electronic games is relatively low, averaging 2.22 hours per week (SD = 5.27). Lastly, the average score on the activation subscale is 21.3 (SD = 6.04).

**Table 3:** Mean and standard deviation of continuous socio-demographic and possible risk factors, along with total DEX-R score for activation elements.

Variable	Mean	±SD
Number of siblings	4.547	1.924
Weight (kg)	65.972	15.025
Height (cm)	167.480	9.806
BMI (calculated)	23.393	4.224
Daily hours spent on social media	5.122	4.105
Weekly hours spent exercising	3.802	5.512
Weekly times eating fast food.	2.104	1.444
Daily hours spent on smartphone/electronic devices	7.082	4.296
Weekly hours spent on electronic games	2.217	5.273
DEX-R activation score	21.299	6.037

**Fig 2:**
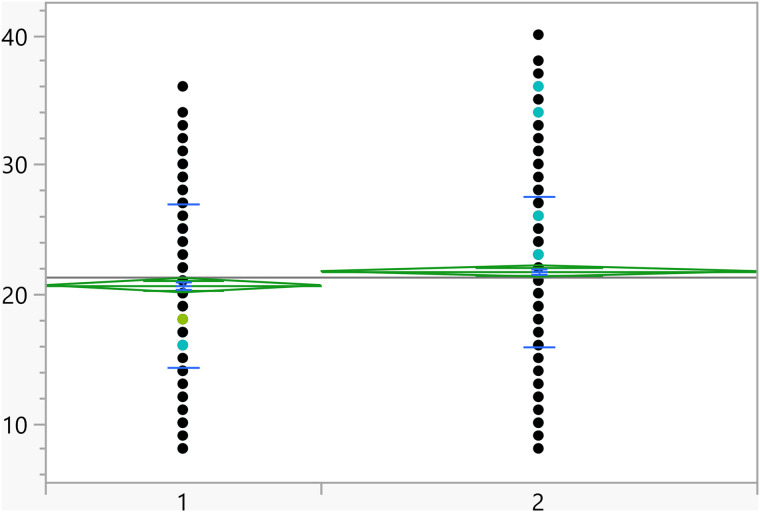
Dot plots of the activation score versus Area of living (1 represent rural). The score was significantly higher for students living in urban areas.

**Fig 3:**
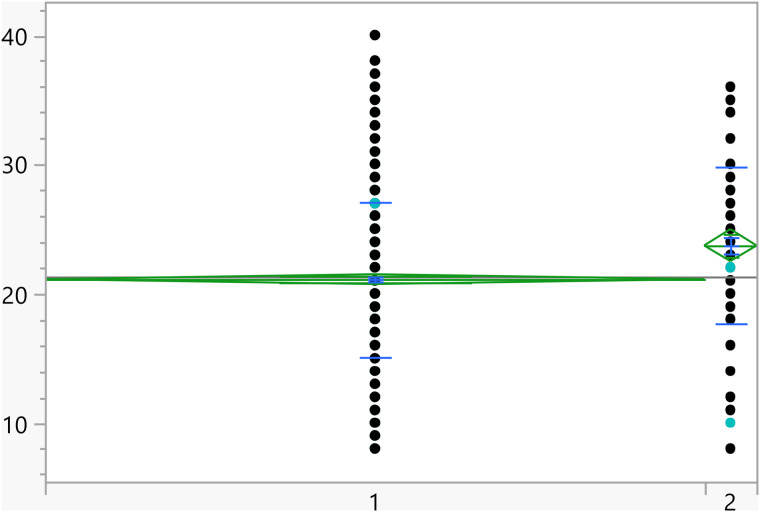
Dot plots of the activation score versus Having visited a psychiatrist/psychologist (1 represent no). Those with prior visits had a higher score on average.

**Fig 4:**
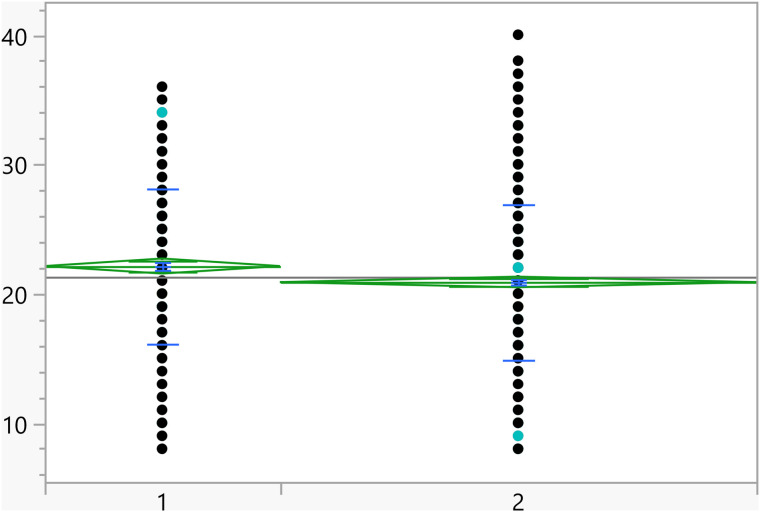
Dot plots of the activation score versus Having strong relationship with extended family (1 represents no, and higher mean score).

**Fig 5:**
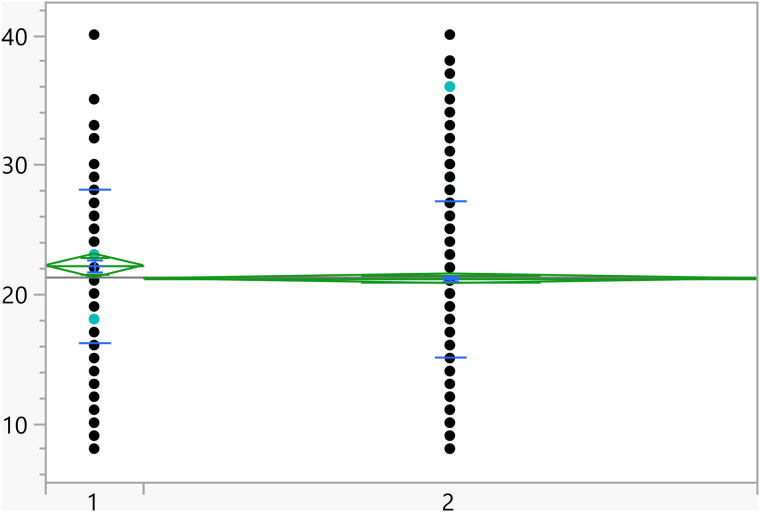
Dot plots of the activation score versus Living with parents (1 represent no, and higher mean score).

**Fig 6:**
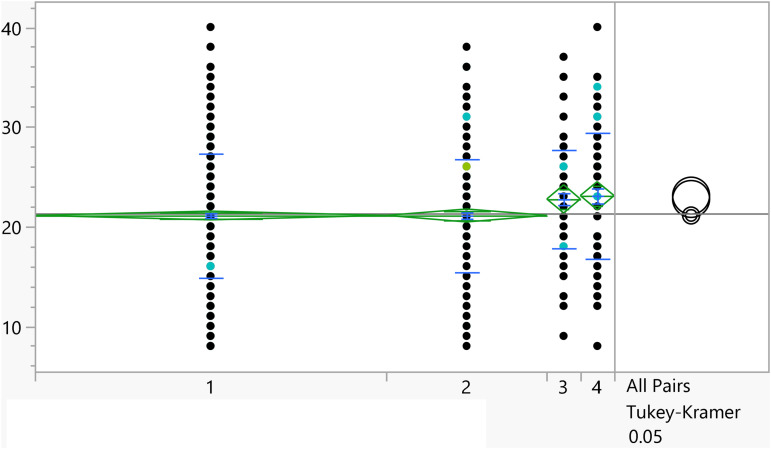
Dot plots of the activation score versus Family income. Significant differences between level 1 and 4. Level with higher income had higher score on average.

**Fig 7:**
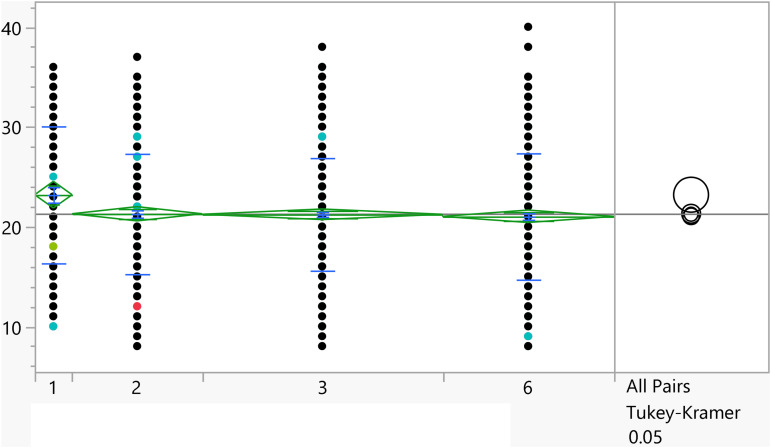
Dot plots of the activation score versus GPA (1 represent <C-), 6 represent (A,A-). Lowest GPA had significantly higher score in comparison to the two highest GPA categories.

**Fig 8:**
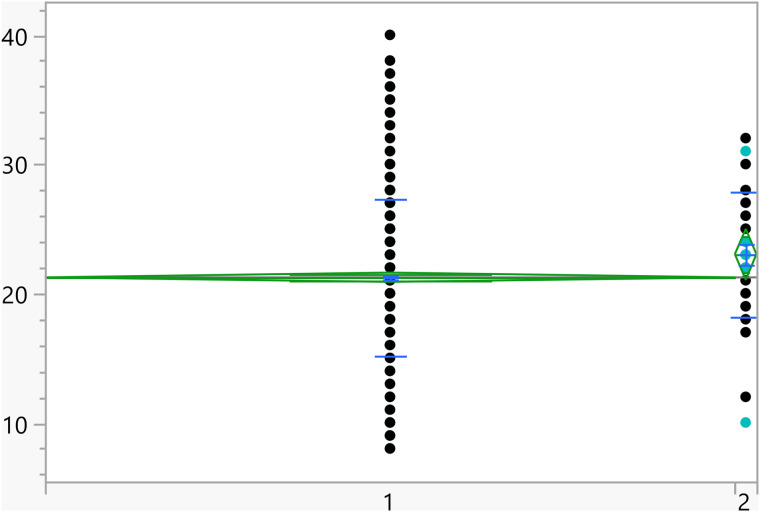
Dot plots of the activation score versus Parents divorced (1 represent no, and lower mean score).

Based on the t-test results, the categorical variables outlined in Table 4 have a significant association with the activation score. Additionally, Figs 2–8 illustrate the distribution of activation scores in relation to these factors. For previous visits to a psychologist or psychiatrist, participants who had prior mental health consultations had a notably higher mean score (23.74 ± 6.05) than those without (21.11 ± 6.00), with a highly significant p-value (<0.0001), suggesting a potential link between mental health support history and activation difficulties. Regarding area of living, urban participants had slightly higher scores (21.72 ± 5.82) than rural residents (20.63 ± 6.32), with a p-value of 0.0023, which could indicate that urban living is associated with modestly increased activation challenges. Family ties also showed a significant association: participants with strong extended family relationships scored lower (20.89 ± 6.02) compared to those without such connections (22.12 ± 6.01), with a p-value of 0.0008, suggesting family support may positively influence activation. Parental marital status was another factor, with students from divorced families showing higher scores (23.02 ± 4.84) than those from intact families (21.25 ± 6.06), marked by a p-value of 0.0406, which could point to potential impacts of parental separation on activation functions. Living arrangements showed that students residing with parents had lower scores (21.16 ± 6.04) compared to those living independently (22.17 ± 5.93), with a p-value of 0.0453, implying that living with parents may benefit activation. Family income was significantly linked to scores (p = 0.0099), where students from families earning above 3K JOD had the highest mean scores (23.07 ± 6.32), while those with lower incomes (under 1K JOD) scored lower on average (21.09 ± 6.23), suggesting financial stability might impact activation. Finally, GPA showed a meaningful relationship with activation (p = 0.0375). Higher-achieving students (A, A-) had the lowest mean scores (21.01 ± 6.33), indicating better activation, while students with GPAs below C- had the highest scores (23.18 ± 6.87), linking academic success to stronger activation. Together, these factors offer insights into how socioeconomic, familial, and academic conditions may shape activation in university students.

**Table 4:** Significant dichotomous and ordinal categorical variables.

Variable	P-value	Value	Mean score	± SD
Previous visits to a psychologist/psychiatrist?	<0.0001	No	21.11	6.00
		Yes	23.74	6.05
Area of living	0.0023	Rural	20.63	6.32
		Urban	21.72	5.82
Strong relationship with extended family?	0.0008	No	22.12	6.01
		Yes	20.89	6.02
Divorced parents?	0.0406	No	21.25	6.06
		Yes	23.02	4.84
Living with parents?	0.0453	No	22.17	5.93
		Yes	21.16	6.04
Family income	0.0099 (levels 1 vs 4)	> 1K	21.09	6.23
		1K-2K	21.09	5.67
		2K-3K	22.74	4.93
		>>3K	23.07	6.32
GPA	0.0375 (highest two levels vs lowest)	<*C*-	23.18	6.87
		C,C-	21.27	6.04
		B,B-	21.22	5.64
		A,A-	21.01	6.33

**Fig 9:**
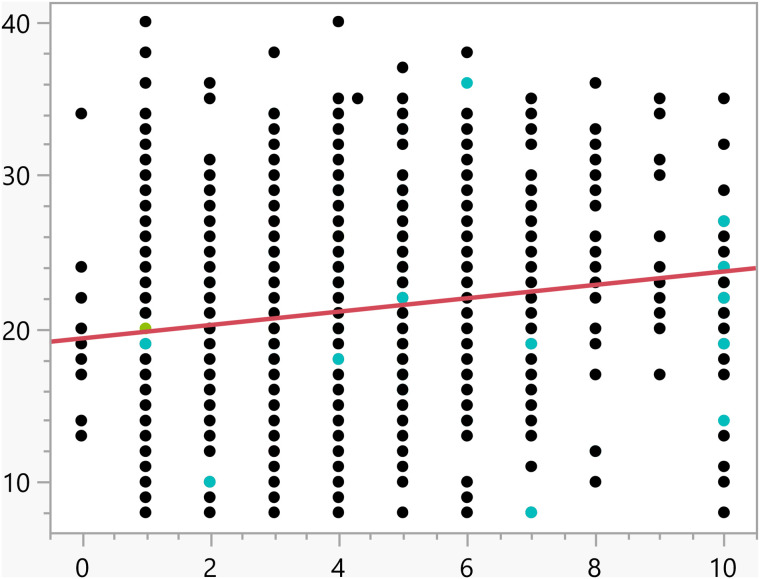
Bivariate fit of the activation score versus Number of daily hours spent on social media.

**Fig 10:**
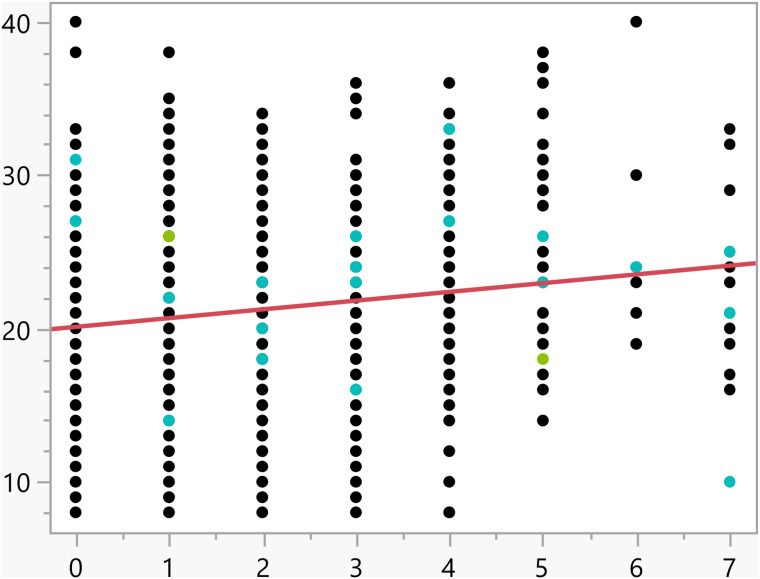
Bivariate fit of the activation score versus Number of weekly times eating fast food.

**Fig 11:**
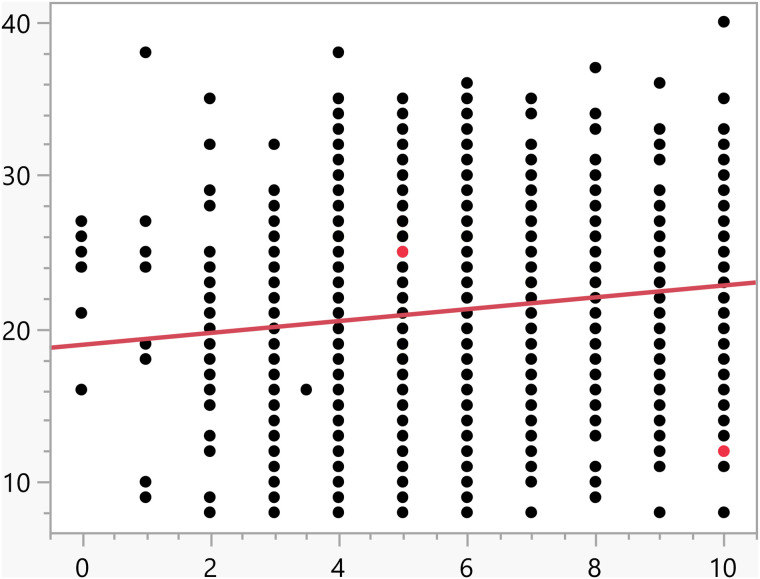
Bivariate fit of the activation score versus Number of daily hours using smartphone/electronic devices.

**Fig 12:**
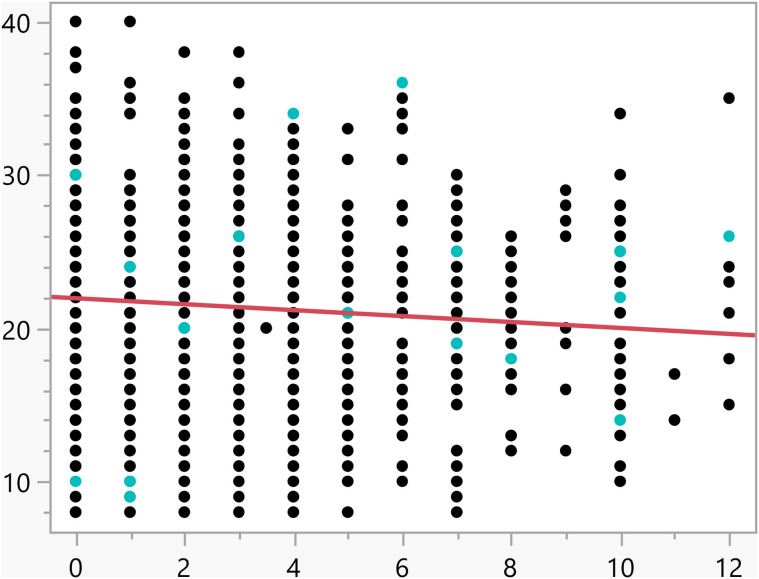
Bivariate fit of the activation score versus Number of weekly hours exercising/playing sports.

**Fig 13:**
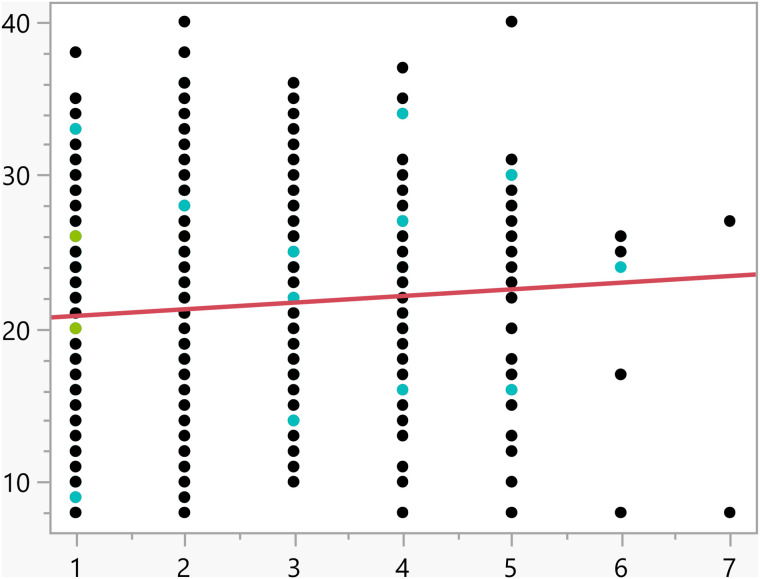
Bivariate fit of the activation score versus Number of social media platforms used.

Table 5 displays the significant continuous factors, their corresponding p-values, and the formulas used for prediction. The bivariate fit of these factors is shown in Figs 9–13. The number of social media platforms used has a significant p-value of 0.0027, suggesting that an increase in the number of platforms correlates with higher activation scores. Similarly, daily hours spent on social media shows a highly significant relationship with a p-value of <0.0001, indicating that more hours spent on social media may lead to elevated activation scores. In contrast, the number of weekly hours exercising or playing sports demonstrates a strong association, with a p-value of <0.0001, suggesting that increased physical activity is linked to improved activation scores. Conversely, the number of weekly times eating fast food shows a significant impact with a p-value of <0.0001, indicating that more frequent fast food consumption correlates with higher activation scores, suggesting a detrimental effect on cognitive activation. Lastly, the number of daily hours using smartphones or electronic devices presents a strong association, with a p-value of <0.0001, indicating that increased screen time is linked to higher activation scores, reflecting potential challenges posed by excessive electronic device use on cognitive functions. Collectively, these factors highlight how lifestyle choices, particularly regarding social media engagement and physical activity, significantly influence activation functions.

**Table 5:** Summary of significant continuous variables. *x* is the corresponding variable in each line.

Variable	P-value	Prediction equation
Number of social media platforms used	0.0027	20.37+0.43×x
Daily hours spent on social media	<0.0001	19.33+0.44×x
Number of weekly hours exercising/playing sports	<0.0001	21.96−0.20×x
Number of weekly times eating fast food	<0.0001	20.10+0.57×x
Number of daily hours using smartphone/electronic devices	<0.0001	18.91+0.39×x

Additional statistical analyses were carried out to identify factors linked to activation dysfunction, focusing on calculating the 25th, 50th, and 75th percentile thresholds. Table 6 presents these cutoff scores, derived from factor analysis, alongside the overall test results. Quartiles are particularly useful in gauging an individual’s relative standing within a group, especially in population-focused studies where variations within groups are essential. In epidemiological research, quartiles facilitate the assessment of risk distribution, enabling researchers to identify outliers or extremes, which can support more tailored interventions. However, while quartiles are valuable for public health studies in pinpointing relative risk and highlighting at-risk subgroups, they may lack precision and can vary across different populations. Total scores, on the other hand, are often more informative for evaluating the absolute severity of activation dysfunction in clinical settings.

**Table 6:** The calculated clinical quartile limits for the activation subscale score. The minimum score was 8 and maximum was 40.

Quartile	Activation score
25% (mild)	17
50% (moderate)	22
75% (strong)	25

Table 7 presents the distribution of activation subscale scores across various demographic and lifestyle groups, categorized into quartiles that reflect differing levels of activation, ranging from Q1 as “normal” to Q4 as “strong” dysfunction or symptoms. Key findings indicate that nationality does not significantly influence activation scores, as Jordanians and non-Jordanians exhibit similar distributions ($\chi^{2}$ = 5.739, p = 0.125). However, area of living shows a significant difference, with rural participants overrepresented in Q3 (“moderate” activation) compared to their urban counterparts ($\chi^{2}$ = 9.352, p = 0.025), suggesting that rural living may affect activation levels. The distribution of activation scores by sex remains consistent, with no significant differences between males and females ($\chi^{2}$ = 5.58, p = 0.1339). Although individuals from divorced families tend to fall into Q4 (“strong” dysfunction), this association is not statistically significant ($\chi^{2}$ = 6.219, p = 0.1014). Participants with stronger relationships with extended family show a more even distribution across quartiles, indicating potential benefits, yet this finding is also not statistically significant ($\chi^{2}$ = 5.036, p = 0.169). Regarding university type, there are no significant differences in activation scores between public and private students, although private university students are somewhat overrepresented in Q4 ($\chi^{2}$ = 5.76, p = 0.1238). A significant association exists between family income and activation levels ($\chi^{2}$ = 19.508, p = 0.0212), with higher-income groups showing more individuals in Q4, implying that financial resources might influence activation. Academic year does not significantly correlate with activation scores ($\chi^{2}$ = 6.55, p = 0.6839), and while students with lower GPAs (<C-) appear slightly more in Q4, this is not statistically significant ($\chi^{2}$ = 7.642, p = 0.5706). Participants who often balance work and study do not show significant differences in activation levels either ($\chi^{2}$ = 8.433, p = 0.4912). The presence of a social media account does not lead to significant variation in activation scores ($\chi^{2}$ = 2.276, p = 0.5172). A significant association is noted for those living with parents, where higher activation is seen in Q2 ($\chi^{2}$ = 16.098, p = 0.0011). Lastly, prior psychological consultations are strongly associated with activation dysfunction ($\chi^{2}$ = 21.9, p < 0.0001), suggesting that those who sought mental health support may be more vulnerable to increased activation dysfunction or symptoms.

**Table 7:** Clinical quartile scores distribution between groups for the activation subscale.

Variable	Value	Q1 Normal	Q2 Mild	Q3 Moderate	Q4 Strong	$\chi^{2}$	P-Value
Nationality	Other	29 (31.2%)	18 (19.4%)	19 (20.4%)	27 (29.0%)	5.739	0.125
	Jordan	302 (27.2%)	231 (20.8%)	249 (22.4%)	329 (29.6%)		
Area of living	Rural	122 (26.1%)	83 (17.8%)	123 (26.3%)	139 (29.8%)	9.352	0.025
	City	209 (28.4%)	166 (22.5%)	145 (19.7%)	217 (29.4%)		
Sex	Male	120 (25.8%)	95 (20.4%)	120 (25.8%)	131 (28.1%)	5.58	0.1339
	Female	211 (28.6%)	154 (20.9%)	148 (20.1%)	225 (30.5%)		
Parents Divorced?	No	320 (27.4%)	239 (20.5%)	266 (22.8%)	343 (29.4%)	6.219	0.1014
	Yes	11 (30.6%)	10 (27.8%)	2 (5.6%)	13 (36.1%)		
Strong relationship with extended family	No	95 (23.9%)	105 (26.4%)	72 (18.1%)	126 (31.7%)	5.036	0.169
	Yes	196 (24.3%)	201 (24.9%)	187 (23.2%)	222 (27.5%)		
University	Private	22 (23.4%)	15 (15.8%)	20 (21.1%)	38 (40.0%)	5.76	0.1238
	Public	309 (27.9%)	234 (21.1%)	248 (22.4%)	318 (28.7%)		
Family income	<1K	193 (26.4%)	140 (19.2%)	180 (24.6%)	218 (29.8%)	19.508	0.0212
	1-2K	102 (30.6%)	73 (21.9%)	71 (21.3%)	87 (26.13%)		
	2-3K	20 (21.4%)	21 (30.0%)	6 (8.6%)	23 (32.9%)		
	>3K	16 (22.9%)	15 (21.43%)	11 (15.7%)	28 (40.0%)		
Undergraduate	Yes	324 (27.3%)	247 (20.8%)	265 (22.3%)	350 (29.5%)	2.017	0.5689
	No	7 (38.9%)	2 (11.11%)	3 (16.7%)	6 (33.3%)		
Academic level (year)	1	25 (25.8%)	19 (19.6%)	24 (24.7%)	29 (29.9%)	6.55	0.6839
	2	167 (27.3%)	123 (20.1%)	137 (22.4%)	185 (30.2%)		
	3	77 (26.2%)	71 (24.2%)	57 (19.4%)	89 (30.3%)		
	4	62 (30.9%)	36 (17.9%)	50 (24.9%)	53 (26.4%)		
GPA	<C-	17 (21.8%)	17 (21.8%)	13 (16.7%)	31 (39.8%)	7.642	0.5706
	C,C-	71 (26.3%)	61 (22.6%)	61 (22.6%)	77 (28.5%)		
	B,B-	143 (28.7%)	105 (21.0%)	107 (21.4%)	144 (28.9%)		
	A,A-	100 (28.0%)	66 (18.5%)	87 (24.4%)	104 (29.1%)		
Work and Study?	Never	224 (28.9%)	155 (20.0%)	166 (21.4%)	231 (29.8%)	8.433	0.4912
	Sometimes	64 (28.6%)	43 (19.2%)	57 (25.5%)	60 (26.8%)		
	Most of the times	21 (22.1%)	23 (24.2%)	20 (21.1%)	31 (32.6%)		
	Always	22 (20.2%)	28 (25.7%)	25 (22.9%)	34 (31.2%)		
Own a social media account?	No	4 (28.6%)	5 (35.7%)	2 (14.3%)	3 (21.4%)	2.276	0.5172
	Yes	327 (27.5%)	244 (20.5%)	266 (22.4%)	353 (29.7%)		
Do you live	No	37 (22.2%)	53 (31.7%)	28 (16.8%)	49 (29.3%)	16.098	0.0011
with parent(s)?	Yes	294 (28.4%)	196 (18.9%)	240 (23.1%)	307 (29.6%)		
Previous visits to a psychologistor or psychiatrist?	No	314 (28.1%)	234 (20.9%)	257 (23.0%)	311 (27.9%)	21.9	<0.0001
	Yes	17 (19.3%)	15 (17.1%)	11 (12.5%)	45 (51.1%)		

## Discussion and conclusions

The growing use of social media and electronic devices among university students has raised concerns about their impact on activation-regulating functions. Activation, which encompasses cognitive processes such as attention, motivation, and emotional regulation, can be significantly influenced by lifestyle choices, including the extent of social media use, smartphone engagement, and dietary habits. In line with the present findings linking smartphone use to deficits in activation-regulating functions, prior evidence indicates that mobile technologies can act as persistent external distractors that undermine students’ capacity to initiate, sustain, and regulate goal-directed behavior, with empirical and review-level studies showing that increased access to smartphones is associated with poorer academic performance due to attentional and self-regulatory disruption and that restricting mobile phone use in educational settings yields measurable improvements in attention, engagement, and learning outcomes [25].

Research indicates that excessive hours spent on social media platforms can lead to heightened levels of anxiety, stress, and depression, which in turn negatively affect activation-regulating functions. For instance, a study by Primack et al. [26] found that higher social media use is correlated with increased feelings of isolation and depressive symptoms among young adults. This relationship can disrupt focus and motivation, essential components of activation, as students may become preoccupied with social comparisons and online interactions rather than their academic responsibilities. Furthermore, the overuse of smartphones and other electronic devices can further exacerbate issues related to activation regulation. A systematic review by Lepp et al. [27] suggests that prolonged screen time is associated with decreased academic performance and lower engagement in educational activities. The constant notifications and distractions from smartphones can fragment attention and reduce the ability to engage deeply with tasks, leading to diminished activation and poorer academic outcomes. Moreover, the number of social media platforms utilized by students has a significant effect on activation functions. According to a study by Kross et al. [28], individuals who engage with multiple platforms are more likely to experience negative emotions and cognitive overload, which can hinder their ability to focus and regulate their activation levels effectively. The cognitive demands of managing various social media accounts can lead to mental fatigue, reducing overall cognitive performance and activation.

Dietary habits, particularly the consumption of junk food, further contribute to the decline in activation-regulating functions. Junk food, characterized by high sugar and fat content, has been linked to cognitive and motivational impairments and mood disturbances. A study by Yau and Potenza [29] demonstrated that diets high in processed foods negatively affect cognitive function and emotional well-being. Poor nutrition can lead to fluctuations in energy levels and mood, impairing the brain’s capacity for effective activation regulation.

In contrast to the negative effects of social media, smartphone use, and poor dietary habits, engaging in regular physical activity has been shown to positively influence activation functions, initiation, and motivation in university students. Exercise and sports participation are associated with numerous psychological and physiological benefits that enhance cognitive functioning and overall well-being. Regular exercise has been linked to improvements in cognitive performance, including enhanced attention, memory, and executive functioning, with a meta-analysis by McAuley and Rudolph [30] indicating that physical activity significantly improves cognitive function, suggesting that exercise can facilitate better activation of cognitive processes. Furthermore, physical activity can foster intrinsic motivation and enhance goal-setting behaviors; a study by Teixeira et al. [31] highlights that exercise not only improves physical health but also promotes a positive self-image and resilience, which are crucial for maintaining motivation in academic settings, leading to better task initiation and persistence in challenging situations. Additionally, exercise is known to reduce stress and anxiety levels, which can otherwise impede activation functions; according to a study by Li et al. [32], physical activity stimulates the release of neurotransmitters, which improve mood and emotional regulation, ultimately enhancing focus and motivation for academic tasks. Lastly, engaging in regular physical activity can increase overall energy levels, making it easier for students to initiate and engage in tasks; a study by Buecker et al. [33] indicates that moderate exercise is associated with increased energy and reduced feelings of fatigue, which are essential for sustaining motivation throughout the academic day.

Several other significant factors can negatively affect activation functions among university students. One such factor is the environment in which students live. Research indicates that living in urban areas, compared to rural settings, can lead to increased stress and cognitive overload due to higher levels of noise, distractions, and social demands. Urban living is associated with elevated psychological stress and reduced cognitive functioning, which can impede students’ ability to focus and maintain motivation [34].

The quality of social relationships also plays a crucial role in activation functions. Students who lack strong relationships with extended family members may experience feelings of isolation and reduced emotional support, both of which are essential for psychological well-being. According to a study by Demetriou [35], strong family connections can provide a buffer against stress and promote better mental health, whereas weak familial ties can exacerbate anxiety and hinder cognitive performance, negatively impacting activation [36].

Another factor is the history of mental health treatment. Students who have previously visited a psychiatrist or psychologist may be dealing with unresolved mental health issues, which can significantly disrupt their activation functions. Research by Kessler et al. [37] shows that mental health challenges such as anxiety and depression are common among students seeking psychological help, and these conditions can severely impair cognitive functioning, motivation, and overall academic performance.

Living arrangements also influence activation functions. Students who do not live with their parents may face additional stressors associated with independent living, such as financial responsibilities and the need to manage daily life without parental support. A study by Forster et al. [38] found that students living away from home often report higher levels of stress and decreased academic performance, which can negatively affect their activation and motivation.

Interestingly, higher income levels can correlate with negative effects on activation. While financial resources can provide advantages, they may also lead to increased expectations and pressure to succeed, which can contribute to stress and anxiety. Research by Luthar and Becker [39] indicates that affluent adolescents often face unique stressors related to societal pressures and parental expectations, which can hinder their motivation and cognitive functioning.

Lastly, lower Grade Point Averages (GPAs) are directly associated with diminished activation functions. Students with lower GPAs often experience a decline in self-esteem and increased anxiety about their academic performance, creating a vicious cycle that negatively impacts their motivation and activation. A study by Yeager and Dweck [40] highlights that academic struggles can lead to decreased motivation and increased fear of failure, further impairing cognitive performance and activation functions.

This study presents several limitations that should be acknowledged. Firstly, including questions regarding bedtime and sleep duration could yield further insights into the factors affecting activation-regulating functions. Secondly, inquiries about preferences for working individually or in teams might provide additional understanding, as individuals facing activation issues may benefit from collaborative environments. Thirdly, future research could strengthen these findings by employing a more diverse sample, especially by comparing students from public and private universities and examining the effects of professional mental health support, such as therapy sessions with psychologists. Fourthly, the number of students from private universities and those who sought mental health services was notably lower than that of their public university counterparts. Fifthly, the reliance on self-reported surveys may introduce biases, as participants might inaccurately represent their behaviors, cognitive processes, and emotional states. To address this concern, the survey was administered online without requiring personal identification, aiming to reduce response bias. Additionally, recognizing that variations in activation functioning might influence task completion, email reminders were sent to encourage participation and lessen the risk of non-response bias. Sixthly, another limitation relates to the wording of specific questions, particularly those concerning smartphone usage, as participants’ estimates of their usage hours may differ significantly, especially when their usage is both frequent and fragmented (e.g., repeatedly checking messages may not result in accurate hourly totals). Seventhly, mental health outcomes were assessed using a limited set of indicators, without detailed measures of social media addiction (e.g., tolerance and dependence) or related behavioral and cognitive outcomes such as concentration difficulties or aggressive behaviors, which should be addressed in future research. Lastly, the GPA range could have included students with D or D- grades as a separate category, potentially offering further insights. Despite these limitations, this study contributes valuable information about the prevalence of activation impairments among university students in Jordan.

## Supporting information

S1 File Output from the statistics software with detailed results. (DOCX)

S2 File Original data. (XLSX)
