# Supplementary material for: Sociodemographic influences on Student Mental Health and their association with activation-regulating functional impairments
Source: PLoS One. 2026 Mar 18;21(3):e0342731. doi: 10.1371/journal.pone.0342731 (PMC12998822; doi:10.1371/journal.pone.0342731)
Supplement: S1 File — (DOCX) [file pone.0342731.s001.docx]

**Distributions**

**Activation**

**Quantiles**

| 100.0% | maximum | 40 |
| --- | --- | --- |
| 99.5% |  | 36.975 |
| 97.5% |  | 33 |
| 90.0% |  | 29 |
| 75.0% | quartile | 25 |
| 50.0% | median | 22 |
| 25.0% | quartile | 17 |
| 10.0% |  | 13 |
| 2.5% |  | 9 |
| 0.5% |  | 8 |
| 0.0% | minimum | 8 |

**Summary Statistics**

| Mean | 21.299834 |
| --- | --- |
| Std Dev | 6.0373429 |
| Std Err Mean | 0.1739933 |
| Upper 95% Mean | 21.641198 |
| Lower 95% Mean | 20.95847 |
| N | 1204 |
| N Missing | 1 |

**Fit Group**

**Oneway Analysis of Activation By Previous visits to a psychologist/therapist.**

**Oneway Anova**

**Summary of Fit**

| Rsquare | 0.012878 |
| --- | --- |
| Adj Rsquare | 0.012057 |
| Root Mean Square Error | 6.000838 |
| Mean of Response | 21.29983 |
| Observations (or Sum Wgts) | 1204 |

**Pooled t Test**

2-1

Assuming equal variances

| Difference | 2.63111 | t Ratio | 3.959927 |
| --- | --- | --- | --- |
| Std Err Dif | 0.66443 | DF | 1202 |
| Upper CL Dif | 3.93469 | Prob > \|t\| | <.0001* |
| Lower CL Dif | 1.32753 | Prob > t | <.0001* |
| Confidence | 0.95 | Prob < t | 1.0000 |

**Analysis of Variance**

| **Source** | **DF** | **Sum of Squares** | **Mean Square** | **F Ratio** | **Prob > F** |
| --- | --- | --- | --- | --- | --- |
| Previous visits to a psychologist/therapist ؟ | 1 | 564.675 | 564.675 | 15.6810 | <.0001* |
| Error | 1202 | 43284.085 | 36.010 |  |  |
| C. Total | 1203 | 43848.760 |  |  |  |

**Means for Oneway Anova**

| **Level** | **Number** | **Mean** | **Std Error** | **Lower 95%** | **Upper 95%** |
| --- | --- | --- | --- | --- | --- |
| 1 | 1116 | 21.1075 | 0.17963 | 20.755 | 21.460 |
| 2 | 88 | 23.7386 | 0.63969 | 22.484 | 24.994 |

Std Error uses a pooled estimate of error variance

**Means and Std Deviations**

| **Level** | **Number** | **Mean** | **Std Dev** | **Std Err Mean** | **Lower 95%** | **Upper 95%** | **Std Dev Lower 95%** | **Std Dev Upper 95%** | **** |
| --- | --- | --- | --- | --- | --- | --- | --- | --- | --- |
| 1 | 1116 | 21.107527 | 5.9970917 | 0.1795182 | 20.755295 | 21.459759 | 5.7582024 | 6.2568134 |  |
| 2 | 88 | 23.738636 | 6.0486425 | 0.6447875 | 22.457051 | 25.020221 | 5.2680415 | 7.1029623 |  |

| Missing Rows | 1 |
| --- | --- |
| Excluded Rows | 5 |

**Oneway Analysis of Activation By Family monthly income**

**Oneway Anova**

**Summary of Fit**

| Rsquare | 0.00942 |
| --- | --- |
| Adj Rsquare | 0.006943 |
| Root Mean Square Error | 6.016346 |
| Mean of Response | 21.29983 |
| Observations (or Sum Wgts) | 1204 |

**Analysis of Variance**

| **Source** | **DF** | **Sum of Squares** | **Mean Square** | **F Ratio** | **Prob > F** |
| --- | --- | --- | --- | --- | --- |
| Family monthly income | 3 | 413.051 | 137.684 | 3.8038 | 0.0099* |
| Error | 1200 | 43435.709 | 36.196 |  |  |
| C. Total | 1203 | 43848.760 |  |  |  |

**Means for Oneway Anova**

| **Level** | **Number** | **Mean** | **Std Error** | **Lower 95%** | **Upper 95%** |
| --- | --- | --- | --- | --- | --- |
| 1 | 731 | 21.0889 | 0.22252 | 20.652 | 21.525 |
| 2 | 333 | 21.0871 | 0.32969 | 20.440 | 21.734 |
| 3 | 70 | 22.7429 | 0.71909 | 21.332 | 24.154 |
| 4 | 70 | 23.0714 | 0.71909 | 21.661 | 24.482 |

Std Error uses a pooled estimate of error variance

**Means and Std Deviations**

| **Level** | **Number** | **Mean** | **Std Dev** | **Std Err Mean** | **Lower 95%** | **Upper 95%** | **Std Dev Lower 95%** | **Std Dev Upper 95%** | **** |
| --- | --- | --- | --- | --- | --- | --- | --- | --- | --- |
| 1 | 731 | 21.088919 | 6.2277792 | 0.2303427 | 20.636706 | 21.541133 | 5.9240788 | 6.5645467 |  |
| 2 | 333 | 21.087087 | 5.6734624 | 0.3109038 | 20.475497 | 21.698677 | 5.2728037 | 6.1405249 |  |
| 3 | 70 | 22.742857 | 4.9334285 | 0.5896575 | 21.566523 | 23.919192 | 4.2300088 | 5.9196511 |  |
| 4 | 70 | 23.071429 | 6.3207077 | 0.7554691 | 21.564309 | 24.578548 | 5.4194865 | 7.584256 |  |

**Means Comparisons**

**Comparisons for all pairs using Tukey-Kramer HSD**

**Confidence Quantile**

| **q*** | **Alpha** |
| --- | --- |
| 2.57270 | 0.05 |

**HSD Threshold Matrix**

Abs(Dif)-HSD

|  | **4** | **3** | **1** | **2** |
| --- | --- | --- | --- | --- |
| 4 | -2.6163 | -2.2877 | 0.0460 | -0.0508 |
| 3 | -2.2877 | -2.6163 | -0.2826 | -0.3794 |
| 1 | 0.0460 | -0.2826 | -0.8096 | -1.0215 |
| 2 | -0.0508 | -0.3794 | -1.0215 | -1.1995 |

Positive values show pairs of means that are significantly different.

**Connecting Letters Report**

| **Level** |  |  | **Mean** |
| --- | --- | --- | --- |
| 4 | A |  | 23.071429 |
| 3 | A | B | 22.742857 |
| 1 |  | B | 21.088919 |
| 2 | A | B | 21.087087 |

Levels not connected by same letter are significantly different.

**Ordered Differences Report**

| **Level** | **- Level** | **Difference** | **Std Err Dif** | **Lower CL** | **Upper CL** | **p-Value** | **** |
| --- | --- | --- | --- | --- | --- | --- | --- |
| 4 | 2 | 1.984341 | 0.791069 | -0.05084 | 4.019524 | 0.0591 |  |
| 4 | 1 | 1.982509 | 0.752734 | 0.04595 | 3.919067 | 0.0425* |  |
| 3 | 2 | 1.655770 | 0.791069 | -0.37941 | 3.690952 | 0.1559 |  |
| 3 | 1 | 1.653938 | 0.752734 | -0.28262 | 3.590496 | 0.1245 |  |
| 4 | 3 | 0.328571 | 1.016948 | -2.28773 | 2.944874 | 0.9883 |  |
| 1 | 2 | 0.001832 | 0.397762 | -1.02149 | 1.025153 | 1.0000 |  |

| Missing Rows | 1 |
| --- | --- |
| Excluded Rows | 5 |

**Oneway Analysis of Activation By strong relationship with extended family**

**Oneway Anova**

**Summary of Fit**

| Rsquare | 0.009246 |
| --- | --- |
| Adj Rsquare | 0.008422 |
| Root Mean Square Error | 6.011866 |
| Mean of Response | 21.29983 |
| Observations (or Sum Wgts) | 1204 |

**Pooled t Test**

2-1

Assuming equal variances

| Difference | -1.2336 | t Ratio | -3.34927 |
| --- | --- | --- | --- |
| Std Err Dif | 0.3683 | DF | 1202 |
| Upper CL Dif | -0.5110 | Prob > \|t\| | 0.0008* |
| Lower CL Dif | -1.9562 | Prob > t | 0.9996 |
| Confidence | 0.95 | Prob < t | 0.0004* |

**Analysis of Variance**

| **Source** | **DF** | **Sum of Squares** | **Mean Square** | **F Ratio** | **Prob > F** |
| --- | --- | --- | --- | --- | --- |
| strong relationship with extended family ؟ | 1 | 405.432 | 405.432 | 11.2176 | 0.0008* |
| Error | 1202 | 43443.328 | 36.143 |  |  |
| C. Total | 1203 | 43848.760 |  |  |  |

**Means for Oneway Anova**

| **Level** | **Number** | **Mean** | **Std Error** | **Lower 95%** | **Upper 95%** |
| --- | --- | --- | --- | --- | --- |
| 1 | 398 | 22.1256 | 0.30135 | 21.534 | 22.717 |
| 2 | 806 | 20.8921 | 0.21176 | 20.477 | 21.308 |

Std Error uses a pooled estimate of error variance

**Means and Std Deviations**

| **Level** | **Number** | **Mean** | **Std Dev** | **Std Err Mean** | **Lower 95%** | **Upper 95%** | **Std Dev Lower 95%** | **Std Dev Upper 95%** | **** |
| --- | --- | --- | --- | --- | --- | --- | --- | --- | --- |
| 1 | 398 | 22.125628 | 6.0053961 | 0.3010233 | 21.533829 | 22.717427 | 5.6151653 | 6.4543629 |  |
| 2 | 806 | 20.89206 | 6.0150545 | 0.2118713 | 20.476174 | 21.307945 | 5.7350647 | 6.3240008 |  |

| Missing Rows | 1 |
| --- | --- |
| Excluded Rows | 5 |

**Oneway Analysis of Activation By area of living**

**Oneway Anova**

**Summary of Fit**

| Rsquare | 0.007717 |
| --- | --- |
| Adj Rsquare | 0.006892 |
| Root Mean Square Error | 6.016503 |
| Mean of Response | 21.29983 |
| Observations (or Sum Wgts) | 1204 |

**Pooled t Test**

2-1

Assuming equal variances

| Difference | 1.08801 | t Ratio | 3.057515 |
| --- | --- | --- | --- |
| Std Err Dif | 0.35585 | DF | 1202 |
| Upper CL Dif | 1.78617 | Prob > \|t\| | 0.0023* |
| Lower CL Dif | 0.38986 | Prob > t | 0.0011* |
| Confidence | 0.95 | Prob < t | 0.9989 |

**Analysis of Variance**

| **Source** | **DF** | **Sum of Squares** | **Mean Square** | **F Ratio** | **Prob > F** |
| --- | --- | --- | --- | --- | --- |
| area of living | 1 | 338.396 | 338.396 | 9.3484 | 0.0023* |
| Error | 1202 | 43510.364 | 36.198 |  |  |
| C. Total | 1203 | 43848.760 |  |  |  |

**Means for Oneway Anova**

| **Level** | **Number** | **Mean** | **Std Error** | **Lower 95%** | **Upper 95%** |
| --- | --- | --- | --- | --- | --- |
| 1 | 467 | 20.6338 | 0.27841 | 20.088 | 21.180 |
| 2 | 737 | 21.7218 | 0.22162 | 21.287 | 22.157 |

Std Error uses a pooled estimate of error variance

**Means and Std Deviations**

| **Level** | **Number** | **Mean** | **Std Dev** | **Std Err Mean** | **Lower 95%** | **Upper 95%** | **Std Dev Lower 95%** | **Std Dev Upper 95%** | **** |
| --- | --- | --- | --- | --- | --- | --- | --- | --- | --- |
| 1 | 467 | 20.633833 | 6.3174915 | 0.2923386 | 20.059368 | 21.208298 | 5.9366504 | 6.7509418 |  |
| 2 | 737 | 21.721845 | 5.8178859 | 0.2143046 | 21.301124 | 22.142566 | 5.5352758 | 6.1311345 |  |

| Missing Rows | 1 |
| --- | --- |
| Excluded Rows | 5 |

**Oneway Analysis of Activation By GPA**

**Oneway Anova**

**Summary of Fit**

| Rsquare | 0.007019 |
| --- | --- |
| Adj Rsquare | 0.004536 |
| Root Mean Square Error | 6.023634 |
| Mean of Response | 21.29983 |
| Observations (or Sum Wgts) | 1204 |

**Analysis of Variance**

| **Source** | **DF** | **Sum of Squares** | **Mean Square** | **F Ratio** | **Prob > F** |
| --- | --- | --- | --- | --- | --- |
| GPA | 3 | 307.763 | 102.588 | 2.8273 | 0.0375* |
| Error | 1200 | 43540.997 | 36.284 |  |  |
| C. Total | 1203 | 43848.760 |  |  |  |

**Means for Oneway Anova**

| **Level** | **Number** | **Mean** | **Std Error** | **Lower 95%** | **Upper 95%** |
| --- | --- | --- | --- | --- | --- |
| 1 | 78 | 23.1795 | 0.68204 | 21.841 | 24.518 |
| 2 | 270 | 21.2741 | 0.36659 | 20.555 | 21.993 |
| 3 | 499 | 21.2244 | 0.26965 | 20.695 | 21.753 |
| 6 | 357 | 21.0140 | 0.31880 | 20.389 | 21.639 |

Std Error uses a pooled estimate of error variance

**Means and Std Deviations**

| **Level** | **Number** | **Mean** | **Std Dev** | **Std Err Mean** | **Lower 95%** | **Upper 95%** | **Std Dev Lower 95%** | **Std Dev Upper 95%** | **** |
| --- | --- | --- | --- | --- | --- | --- | --- | --- | --- |
| 1 | 78 | 23.179487 | 6.867472 | 0.7775881 | 21.631112 | 24.727863 | 5.9332215 | 8.1536686 |  |
| 2 | 270 | 21.274074 | 6.0357419 | 0.3673236 | 20.550879 | 21.997269 | 5.5659621 | 6.5928052 |  |
| 3 | 499 | 21.224449 | 5.6406552 | 0.2525104 | 20.728332 | 21.720566 | 5.3110626 | 6.0141931 |  |
| 6 | 357 | 21.014006 | 6.3300891 | 0.3350238 | 20.355131 | 21.67288 | 5.897323 | 6.831928 |  |

**Means Comparisons**

**Comparisons for all pairs using Tukey-Kramer HSD**

**Confidence Quantile**

| **q*** | **Alpha** |
| --- | --- |
| 2.57270 | 0.05 |

**HSD Threshold Matrix**

Abs(Dif)-HSD

|  | **1** | **2** | **3** | **6** |
| --- | --- | --- | --- | --- |
| 1 | -2.4815 | -0.0867 | 0.0682 | 0.2286 |
| 2 | -0.0867 | -1.3338 | -1.1212 | -0.9898 |
| 3 | 0.0682 | -1.1212 | -0.9811 | -0.8638 |
| 6 | 0.2286 | -0.9898 | -0.8638 | -1.1599 |

Positive values show pairs of means that are significantly different.

**Connecting Letters Report**

| **Level** |  |  | **Mean** |
| --- | --- | --- | --- |
| 1 | A |  | 23.179487 |
| 2 | A | B | 21.274074 |
| 3 |  | B | 21.224449 |
| 6 |  | B | 21.014006 |

Levels not connected by same letter are significantly different.

**Ordered Differences Report**

| **Level** | **- Level** | **Difference** | **Std Err Dif** | **Lower CL** | **Upper CL** | **p-Value** | **** |
| --- | --- | --- | --- | --- | --- | --- | --- |
| 1 | 6 | 2.165482 | 0.7528731 | 0.22857 | 4.102398 | 0.0213* |  |
| 1 | 3 | 1.955038 | 0.7334135 | 0.06819 | 3.841891 | 0.0389* |  |
| 1 | 2 | 1.905413 | 0.7743174 | -0.08667 | 3.897499 | 0.0667 |  |
| 2 | 6 | 0.260068 | 0.4858211 | -0.98980 | 1.509940 | 0.9504 |  |
| 3 | 6 | 0.210443 | 0.4175525 | -0.86379 | 1.284680 | 0.9581 |  |
| 2 | 3 | 0.049625 | 0.4550819 | -1.12116 | 1.220414 | 0.9995 |  |

| Missing Rows | 1 |
| --- | --- |
| Excluded Rows | 5 |

**Oneway Analysis of Activation By living with parents**

**Oneway Anova**

**Summary of Fit**

| Rsquare | 0.00333 |
| --- | --- |
| Adj Rsquare | 0.002501 |
| Root Mean Square Error | 6.029788 |
| Mean of Response | 21.29983 |
| Observations (or Sum Wgts) | 1204 |

**Pooled t Test**

2-1

Assuming equal variances

| Difference | -1.0076 | t Ratio | -2.00408 |
| --- | --- | --- | --- |
| Std Err Dif | 0.5028 | DF | 1202 |
| Upper CL Dif | -0.0212 | Prob > \|t\| | 0.0453* |
| Lower CL Dif | -1.9940 | Prob > t | 0.9774 |
| Confidence | 0.95 | Prob < t | 0.0226* |

**Analysis of Variance**

| **Source** | **DF** | **Sum of Squares** | **Mean Square** | **F Ratio** | **Prob > F** |
| --- | --- | --- | --- | --- | --- |
| living with parents ؟ | 1 | 146.027 | 146.027 | 4.0163 | 0.0453* |
| Error | 1202 | 43702.733 | 36.358 |  |  |
| C. Total | 1203 | 43848.760 |  |  |  |

**Means for Oneway Anova**

| **Level** | **Number** | **Mean** | **Std Error** | **Lower 95%** | **Upper 95%** |
| --- | --- | --- | --- | --- | --- |
| 1 | 167 | 22.1677 | 0.46660 | 21.252 | 23.083 |
| 2 | 1037 | 21.1601 | 0.18725 | 20.793 | 21.527 |

Std Error uses a pooled estimate of error variance

**Means and Std Deviations**

| **Level** | **Number** | **Mean** | **Std Dev** | **Std Err Mean** | **Lower 95%** | **Upper 95%** | **Std Dev Lower 95%** | **Std Dev Upper 95%** | **** |
| --- | --- | --- | --- | --- | --- | --- | --- | --- | --- |
| 1 | 167 | 22.167665 | 5.9309812 | 0.4589531 | 21.261527 | 23.073802 | 5.3558283 | 6.6456151 |  |
| 2 | 1037 | 21.160077 | 6.0454702 | 0.187733 | 20.791697 | 21.528458 | 5.7960177 | 6.3175267 |  |

| Missing Rows | 1 |
| --- | --- |
| Excluded Rows | 5 |

**Oneway Analysis of Activation By parents divorced?**

**Oneway Anova**

**Summary of Fit**

| Rsquare | 0.002527 |
| --- | --- |
| Adj Rsquare | 0.001697 |
| Root Mean Square Error | 6.032218 |
| Mean of Response | 21.29983 |
| Observations (or Sum Wgts) | 1204 |

**Pooled t Test**

2-1

Assuming equal variances

| Difference | 1.7812 | t Ratio | 1.745001 |
| --- | --- | --- | --- |
| Std Err Dif | 1.0207 | DF | 1202 |
| Upper CL Dif | 3.7838 | Prob > \|t\| | 0.0812 |
| Lower CL Dif | -0.2214 | Prob > t | 0.0406* |
| Confidence | 0.95 | Prob < t | 0.9594 |

**Analysis of Variance**

| **Source** | **DF** | **Sum of Squares** | **Mean Square** | **F Ratio** | **Prob > F** |
| --- | --- | --- | --- | --- | --- |
| Parents divorced؟ | 1 | 110.801 | 110.801 | 3.0450 | 0.0812 |
| Error | 1202 | 43737.959 | 36.388 |  |  |
| C. Total | 1203 | 43848.760 |  |  |  |

**Means for Oneway Anova**

| **Level** | **Number** | **Mean** | **Std Error** | **Lower 95%** | **Upper 95%** |
| --- | --- | --- | --- | --- | --- |
| 1 | 1168 | 21.2466 | 0.1765 | 20.900 | 21.593 |
| 2 | 36 | 23.0278 | 1.0054 | 21.055 | 25.000 |

Std Error uses a pooled estimate of error variance

**Means and Std Deviations**

| **Level** | **Number** | **Mean** | **Std Dev** | **Std Err Mean** | **Lower 95%** | **Upper 95%** | **Std Dev Lower 95%** | **Std Dev Upper 95%** | **** |
| --- | --- | --- | --- | --- | --- | --- | --- | --- | --- |
| 1 | 1168 | 21.246575 | 6.0642791 | 0.1774425 | 20.898433 | 21.594717 | 5.8279416 | 6.3207423 |  |
| 2 | 36 | 23.027778 | 4.8431755 | 0.8071959 | 21.389083 | 24.666473 | 3.9282105 | 6.3176224 |  |

| Missing Rows | 1 |
| --- | --- |
| Excluded Rows | 5 |

**Oneway Analysis of Activation By University**

**Oneway Anova**

**Summary of Fit**

| Rsquare | 0.002428 |
| --- | --- |
| Adj Rsquare | 0.001598 |
| Root Mean Square Error | 6.032518 |
| Mean of Response | 21.29983 |
| Observations (or Sum Wgts) | 1204 |

**Pooled t Test**

2-1

Assuming equal variances

| Difference | -1.1030 | t Ratio | -1.71035 |
| --- | --- | --- | --- |
| Std Err Dif | 0.6449 | DF | 1202 |
| Upper CL Dif | 0.1622 | Prob > \|t\| | 0.0875 |
| Lower CL Dif | -2.3682 | Prob > t | 0.9563 |
| Confidence | 0.95 | Prob < t | 0.0437* |

**Analysis of Variance**

| **Source** | **DF** | **Sum of Squares** | **Mean Square** | **F Ratio** | **Prob > F** |
| --- | --- | --- | --- | --- | --- |
| university | 1 | 106.455 | 106.455 | 2.9253 | 0.0875 |
| Error | 1202 | 43742.304 | 36.391 |  |  |
| C. Total | 1203 | 43848.760 |  |  |  |

**Means for Oneway Anova**

| **Level** | **Number** | **Mean** | **Std Error** | **Lower 95%** | **Upper 95%** |
| --- | --- | --- | --- | --- | --- |
| 1 | 95 | 22.3158 | 0.61892 | 21.101 | 23.530 |
| 2 | 1109 | 21.2128 | 0.18115 | 20.857 | 21.568 |

Std Error uses a pooled estimate of error variance

**Means and Std Deviations**

| **Level** | **Number** | **Mean** | **Std Dev** | **Std Err Mean** | **Lower 95%** | **Upper 95%** | **Std Dev Lower 95%** | **Std Dev Upper 95%** | **** |
| --- | --- | --- | --- | --- | --- | --- | --- | --- | --- |
| 1 | 95 | 22.315789 | 6.722878 | 0.6897527 | 20.946269 | 23.68531 | 5.8839658 | 7.842992 |  |
| 2 | 1109 | 21.212804 | 5.9702767 | 0.1792787 | 20.86104 | 21.564568 | 5.7317358 | 6.2296879 |  |

| Missing Rows | 1 |
| --- | --- |
| Excluded Rows | 5 |

**Bivariate Fit of Activation By social media platforms used most of the times**

**Linear Fit**

Activation = 20.368517 + 0.4344459* social media platforms used most of the times) ؟

**Summary of Fit**

| RSquare | 0.007482 |
| --- | --- |
| RSquare Adj | 0.006656 |
| Root Mean Square Error | 6.017217 |
| Mean of Response | 21.29983 |
| Observations (or Sum Wgts) | 1204 |

**Analysis of Variance**

| **Source** | **DF** | **Sum of Squares** | **Mean Square** | **F Ratio** |
| --- | --- | --- | --- | --- |
| Model | 1 | 328.063 | 328.063 | 9.0608 |
| Error | 1202 | 43520.697 | 36.207 | **Prob > F** |
| C. Total | 1203 | 43848.760 |  | 0.0027* |

**Parameter Estimates**

| **Term** | **Estimate** | **Std Error** | **t Ratio** | **Prob>\|t\|** |
| --- | --- | --- | --- | --- |
| Intercept | 20.368517 | 0.35468 | 57.43 | <.0001* |
| social media platforms used most of the times ؟ | 0.4344459 | 0.144329 | 3.01 | 0.0027* |

**Bivariate Fit of Activation By daily hours using social media**

**Linear Fit**

Activation = 19.334993 + 0.4374938* daily hours using social media ؟

**Summary of Fit**

| RSquare | 0.02521 |
| --- | --- |
| RSquare Adj | 0.024347 |
| Root Mean Square Error | 5.989606 |
| Mean of Response | 21.23852 |
| Observations (or Sum Wgts) | 1132 |

**Analysis of Variance**

| **Source** | **DF** | **Sum of Squares** | **Mean Square** | **F Ratio** |
| --- | --- | --- | --- | --- |
| Model | 1 | 1048.427 | 1048.43 | 29.2241 |
| Error | 1130 | 40539.174 | 35.88 | **Prob > F** |
| C. Total | 1131 | 41587.601 |  | <.0001* |

**Parameter Estimates**

| **Term** | **Estimate** | **Std Error** | **t Ratio** | **Prob>\|t\|** |
| --- | --- | --- | --- | --- |
| Intercept | 19.334993 | 0.394561 | 49.00 | <.0001* |
| Daily hours using social media | 0.4374938 | 0.080928 | 5.41 | <.0001* |

**Bivariate Fit of Activation By weekly hours excercising**

**Linear Fit**

Activation = 21.955498 - 0.1953959*weekly hours excecising

**Summary of Fit**

| RSquare | 0.007941 |
| --- | --- |
| RSquare Adj | 0.007077 |
| Root Mean Square Error | 6.075364 |
| Mean of Response | 21.36957 |
| Observations (or Sum Wgts) | 1150 |

**Analysis of Variance**

| **Source** | **DF** | **Sum of Squares** | **Mean Square** | **F Ratio** |
| --- | --- | --- | --- | --- |
| Model | 1 | 339.197 | 339.197 | 9.1898 |
| Error | 1148 | 42372.738 | 36.910 | **Prob > F** |
| C. Total | 1149 | 42711.935 |  | 0.0025* |

**Parameter Estimates**

| **Term** | **Estimate** | **Std Error** | **t Ratio** | **Prob>\|t\|** |
| --- | --- | --- | --- | --- |
| Intercept | 21.955498 | 0.263542 | 83.31 | <.0001* |
| Weekly hours excercising | -0.195396 | 0.064456 | -3.03 | 0.0025* |

**Bivariate Fit of Activation By ؟weekly times eating fast food**

**Linear Fit**

Activation = 20.098746 + 0.5711336* weekly times eating fast food

**Summary of Fit**

| RSquare | 0.018637 |
| --- | --- |
| RSquare Adj | 0.01782 |
| Root Mean Square Error | 5.983308 |
| Mean of Response | 21.29983 |
| Observations (or Sum Wgts) | 1204 |

**Analysis of Variance**

| **Source** | **DF** | **Sum of Squares** | **Mean Square** | **F Ratio** |
| --- | --- | --- | --- | --- |
| Model | 1 | 817.190 | 817.190 | 22.8265 |
| Error | 1202 | 43031.570 | 35.800 | **Prob > F** |
| C. Total | 1203 | 43848.760 |  | <.0001* |

**Parameter Estimates**

| **Term** | **Estimate** | **Std Error** | **t Ratio** | **Prob>\|t\|** |
| --- | --- | --- | --- | --- |
| Intercept | 20.098746 | 0.304849 | 65.93 | <.0001* |
| weekly times eating fast food | 0.5711336 | 0.119541 | 4.78 | <.0001* |

**Bivariate Fit of Activation By daily hours using smart devices**

**Linear Fit**

Activation = 18.914364 + 0.387596* daily hours using smart devices

**Summary of Fit**

| RSquare | 0.019949 |
| --- | --- |
| RSquare Adj | 0.019011 |
| Root Mean Square Error | 5.969307 |
| Mean of Response | 21.16157 |
| Observations (or Sum Wgts) | 1046 |

**Analysis of Variance**

| **Source** | **DF** | **Sum of Squares** | **Mean Square** | **F Ratio** |
| --- | --- | --- | --- | --- |
| Model | 1 | 757.235 | 757.235 | 21.2512 |
| Error | 1044 | 37200.460 | 35.633 | **Prob > F** |
| C. Total | 1045 | 37957.695 |  | <.0001* |

**Parameter Estimates**

| **Term** | **Estimate** | **Std Error** | **t Ratio** | **Prob>\|t\|** |
| --- | --- | --- | --- | --- |
| Intercept | 18.914364 | 0.521245 | 36.29 | <.0001* |
| Daily hours using smart devices | 0.387596 | 0.084079 | 4.61 | <.0001* |
